# Growth, health, and economics of dairy calves fed organic milk replacer or organic whole milk in an automated feeding system

**DOI:** 10.3168/jdsc.2021-0084

**Published:** 2021-08-26

**Authors:** K.T. Sharpe, B.J. Heins

**Affiliations:** Department of Animal Science, University of Minnesota, St. Paul 55108

## Abstract

The objective of the study was to investigate the growth, health, behavior, and economics of dairy calves fed organic milk replacer (n = 41) or organic whole milk (n = 40) in an automatic feeding system. Calves were fed either organic milk replacer or whole milk (assigned to treatment in birth order) during 2 seasons from March to July 2018 and from September to December 2018 at the University of Minnesota West Central Research and Outreach Center in Morris, Minnesota. The treatment groups were (1) pasteurized whole milk fed at 13% total solids of organic milk (WM), or (2) milk replacer fed at 150.98 g of dry replacer powder per liter of water (MR). Milk replacer was fed at 14.65% total solids based on the manufacturer's recommendation. Calves were introduced to the automated feeder at 5 d and allowed to drink up to 8 L/d at the maximum allowance. At 50 d, the allowance was reduced by 0.2 L/d and calves were weaned at 56 d. Milk feeding behavior (feeding station visit behaviors and drinking speeds) were collected from the automatic feeding system and analyzed by feeding group. Body weights were recorded at birth and then weekly until weaning (56 d). Health scores of calves were recorded twice a week. Data were analyzed using PROC MIXED (SAS Institute Inc.). Independent variables for analyses were the fixed effects of breed group, season of birth, and treatment group, and the interaction of season and treatment group along with pen as a random effect. No differences were found between treatment groups for average daily gain, weaning weight, hip height, or heart girth. Milk feeding behavior varied between the 2 feeding treatment groups. The WM calves had shorter visits to the feeding station (2.44 vs. 3.01 min, respectively) compared with MR calves. Overall drinking speeds of the WM calves were higher (1,301 mL/min) than those of the MR calves (581 mL/min). The MR calves had higher fecal scores than WM calves. The average cost per kilogram of gain was lower for WM calves ($6.35/kg) compared with MR calves ($8.82/kg). The results of this study indicate health and economic advantages to feeding organic dairy heifer calves whole milk during the preweaning period.

## Highlights

• Organic milk replacer was not available for organic dairy farmers until recently.
• Calves had similar growth whether they were fed organic milk replacer or organic whole milk.
• Milk feed cost was greater for calves fed organic milk replacer because of its higher cost.
• Organic milk replacer may be used for convenience on organic dairy farms.

## Graphical Abstract

**Figure:**
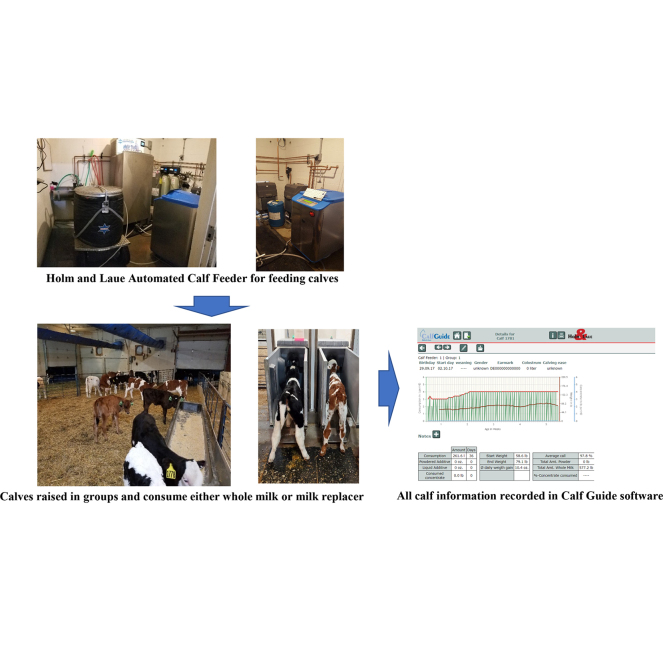
**Summary:** In this study, we compared the growth, health, behavior, and economics of feeding dairy calves organic milk replacer or whole milk during the preweaning period. Calves fed organic milk replacer had similar growth to calves fed organic whole milk. Milk feed cost was 37% lower for calves fed organic whole milk. The average cost per kilogram of gain was lower for calves fed organic whole milk compared with calves fed organic milk replacer. Organic dairy producers may find economic advantages of feeding whole milk to organic dairy heifer calves during the preweaning period compared with organic milk replacer because of the high cost.

Organic and conventional management of raising replacement dairy heifers is an expensive and important investment for a dairy operation (Heinrichs et al., 2013). Growing replacement heifers accounts for 25% of the total cost of milk production and is the second largest expense for a dairy after feed costs (Zwald et al., 2007). The nutrition, health, and management of replacements may have a major impact on the profitability of the entire dairy operation. Feeding of organic dairy calves requires different management because calves require certified organic feed, animals must have year-round access to the outdoors, and organic management does not allow for use of antibiotics unless to restore animal health when organic methods fail (USDA-AMS, 2021). Recent studies with organic dairy calves have reported that the average cost per gain of organic dairy calves ranges from $3.02/kg to $4.13/kg (Bjorklund et al., 2013; Kienitz et al., 2017). Organic producers, especially, may be faced with challenges such as higher feed costs and maintaining animal health. Therefore, it is essential that organic producers manage feed costs and profitability while also rearing healthy calves.

Selecting an appropriate liquid feeding program is an important aspect of nutritional management and plays a major role in the health of preweaning dairy calves (Godden et al., 2005). Feed costs for preweaning organic heifer calves typically include the cost of raw or pasteurized nonsalable or saleable organic whole milk (or a combination of both) and organic calf starter grain. Until recently, organic dairy producers did not have an option to feed calves milk replacer because no organic milk replacer was available on the market. However, an organic milk replacer (Organi-Calf Instant Milk, Milk Specialties Global) was approved by the USDA for use in organic systems in the fall of 2017.

No studies have compared feeding organic whole milk to organic milk replacer for growth and profitability. Additionally, no studies have utilized an automated feeding system to compare the growth and health of calves fed whole milk versus milk replacer. Therefore, the objective of the study was to determine growth, health, and profitability of dairy calves fed pasteurized organic whole milk (a combination of saleable and nonsaleable) compared with an organic milk replacer in an automated calf feeding production system. Furthermore, milk feeding behaviors from the automated feeding program were compared. Our hypothesis was that calves fed organic whole milk would have greater rates of gain, fewer health challenges, and lower cost of production than calves fed organic milk replacer.

This study was conducted at the University of Minnesota West Central Research and Outreach Center in Morris, Minnesota. All animal procedures involving animal care and management were approved by the University of Minnesota Institutional Animal Care and Use Committee (#1708B11841). Data were from 81 organic dairy heifer calves in 2 calving seasons: 41 heifer calves born from March 24 to May 31, 2018, and 40 heifer calves born from September 11 to October 13, 2018. All calves were born at the University of Minnesota organic dairy research herd (Morris, MN). Breed groups of calves were Holsteins (n = 18; 8 whole milk and 10 milk replacer), including animals with 1964 genetics from a University of Minnesota control population design as described in Hansen (2000) and contemporary Holstein genetics; crossbreds (n = 44; 21 whole milk and 23 milk replacer), including combinations of Holstein, Montbéliarde, and Viking Red; and crossbreds (n = 19; 11 whole milk and 8 milk replacer) including combinations of Jersey, Viking Red, and Normande.

Calves were separated from their dams at birth, moved to indoor housing in individual pens (Calf-Tel I-Series 22|64 1.83 m pen; Calf-Tel, Hampel Corp.), and fed 1.89 L of colostrum per 41 kg of BW 2 times/d (3.78 L/d in total) for 4 d. Colostrum was fed from the dams of calves and was not pasteurized. Calves were moved to the automated group feeding pens at 5 d of age and assigned to 1 of 2 feeding treatments: milk replacer (**MR**) or whole milk (**WM**) based on birth order. The experiment started with a flip of a coin to determine the starting treatment. The MR treatment was selected first, and thereafter, calves alternated between WM and MR treatments. The first pen of calves was formed (21 calves in the spring, 20 calves in the fall) before the second pen of calves was formed (20 calves in the spring and 20 calves in the fall). The time for both pens (41 total calves) to form in spring 2018 was 68 d. The time for both pens (40 total calves) to form in fall 2018 was 32 d. The time for group formation was different between spring and fall because more cows in the research dairy herd calve during the fall compared with the spring. Sex ratios of calves may have affected group time formation. Two pens of calves were formed during the spring and 2 pens of calves were formed during the fall. The calf barn at the research herd that housed the automated feeder had 2 pens that had space for 21 calves. Therefore, 2 pens of calves per season were used for calves. Both MR and WM calves were raised together in all pens (10 calves fed WM and 10 calves fed MR were in the same group of 20 calves). A group of 20 or 21 calves was formed before the next group of calves was formed. Each automated feeding pen had an indoor area of 12.2 × 4.9 m bedded with organic wheat straw and access to an outdoor area that measured 10.7 × 4.9 m and had a small bedded pack of wheat straw.

Whole milk calves (n = 40) were fed at 13% total solids of pasteurized saleable and nonsaleable organic milk. The organic milk averaged 4.2% fat, 3.3% protein, and 5.5% other solids. On a DM basis, the WM contained 32.3% fat, 26.8% protein, 102,168 IU/kg vitamin A, 2,655 IU/kg vitamin D_3_, and 76 IU/kg vitamin E. Milk replacer (n = 41) were fed at 285.76 g of dry milk replacer in 1.89 L (150.98 g/L), based on the manufacturer's suggested feeding rate (Organi-Calf Instant Milk, Milk Specialties Global). The MR was fed at 14.65% total solids. The milk replacer was a milk protein and whey formula and contained 20% CP, 22% fat, 0.15% fiber, 0.99% calcium, 66,138 IU/kg vitamin A, 22,046 IU/kg vitamin D_3_, and 440.9 IU/kg vitamin E.

Free-choice texturized calf starter and water were provided when calves were moved to the automated group feeding pens. Calf starter was 19% CP from organic corn, wheat, expelled soybean meal, soybean oil, and minerals. The calf starter was mixed on site at the research dairy and contained (as a percentage of DM) 89.9% DM, 19.4% CP, 19.2% NDF, 5.94% crude fat, 5.9% ash, 1.52% calcium, 0.74% phosphorus, 50.2% NFC, 47.1% starch, and 0.13 MJ of ME/kg. Individual starter consumption was not recorded because calves were group housed in the automatic milk feeding pens.

The Holm & Laue HL100 Programmable Calf Feeder (Holm & Laue GmbH & Co KG) had the ability to dispense both WM and MR from the same machine, and only one machine was used to feed both treatments. Each pen of calves had 2 nipple feeding stations, for a total of 4 nipple feeding stations. However, only 1 feeding station per pen was allowed to feed a calf at a time because of software limitations of the HL100 feeder. Only 2 calves (1 per pen of 20 calves) were allowed to drink at any time because the HL100 feeder did not allow calves to drink from all 4 nipple feeding stations at one time. If any WM or MR was left in the feeding bowl from a calf, the machine discarded the WM or MR before another calf was allowed to feed. A warm-water rinse of the mixing bowl was completed by the feeder after each calf visit where milk was fed. From 5 to 11 d of age, calves in both feeding treatments were on a “ramp-up” phase where feeding amounts were increased from 6 to 8 L/d in 0.2-L daily increments. From 12 to 49 d of age, calves were on a “hold” phase of 8 L/d of peak allowance. From 50 d to weaning at 56 d, calves transitioned to a “ramp-down” phase where total allowance was decreased from 8 to 6 L/d in 0.2-L increments. Meals to calves were delivered in 2.2-L increments. If a calf drank all 2.2 L, they were not allowed to received more milk until 2 h later. Calves were allowed to drink 4 L per half day and a total of 8 L/d. Calves were allowed into the nipple feeding station as many times per day as they wanted to visit; however, milk was not always dispensed based on requirements for calves set in the software. The settings for both treatments were the same, and WM or MR and milk were pumped to the teat by a peristaltic pump in the nipple feeding station. Each individual calf has its own drinking speed.

Body measurements of individual calves included birth weight, weaning weight, weaning hip height, weaning heart girth, total gain, and ADG. Total weight gain was final weaning weight minus calf birth weight. The ADG was birth weight subtracted from weaning weight divided by 56 d. Mortality records and health treatments were documented on an individual calf basis. Individual health scores were recorded on all calves twice per week. The health scoring method was adapted from McGuirk (2018), and calf health was scored using physical indicators on a 0 to 4 scale. A fecal score of 0 represented normal and healthy, and a score of 4 represented a severely abnormal and unhealthy calf.

Milk feeding behaviors were analyzed for all 81 calves on the study, which represented a total of 4,131 calf days. Milk feeding behaviors were recorded by the Holm and Laue CalfGuide automated feeder software (CalfGuide, Holm & Laue GmbH & Co KG) and downloaded to Excel (Microsoft Corp.) for analysis. Milking feeding behaviors included feeding station visit duration (min) and overall average drinking speed (mL/min).

Total health cost was determined from the actual costs of treatments administered to calves. Total milk feed cost was a function of the total cost for organic whole milk or organic milk replacer for an individual calf to weaning. The default milk price was $0.61/kg, which was the mean organic mailbox milk price from March to July 2018 and from September to December 2018 for the research organic dairy. The milk replacer was $6.61/kg. Average cost per day was the sum of health cost and milk feed cost per calf divided by days on the automated feeder (56 d). Average cost per kilogram of gain was the sum of health and milk feed costs divided by the total weight gain for individual calves. Milk intake was the total milk intake by each calf on a DM basis. Gain-to-feed ratio was the sum of kilograms of gain from 56 d divided by the total kilograms of milk consumed on a DM basis, and feed-to-gain ratio was kilograms of milk consumed divided by total gain.

For all measurements, the MIXED procedure (SAS Institute, 2018) was used to obtain solutions and conduct the ANOVA. All treatment results were reported as least squares means with significance declared at *P* < 0.05. For statistical analysis of birth weight, weaning weight, hip height at weaning, heart girth at weaning, total gain, ADG, kilograms of milk intake on a DM basis, fecal score, health cost, milk feed cost, average cost per day, and average cost per gain, gain-to-feed ratio, and feed-to-gain ratio, the independent variables were the fixed effects of breed group, season of birth, and treatment group (WM or MR), and the interaction of season and treatment group, along with pen as a random effect. Calf birth weight was a covariate in the statistical model. Fecal scores were averaged by calf for analyses. Calf was the experimental unit for all analyses. For analysis of visit duration and drinking speed, the independent variables were the fixed effects of treatment, breed, season of birth, and days within treatment group, and the interaction of treatment within season of birth. Random effects were pen and calf within pen, with day as repeated measures. The model that resulted in the lowest Akaike information criterion for repeated measures with the compound symmetry covariance structure (Littell et al., 1998) was used. Individual calf was used as the experimental unit for all statistical analyses.

Calf birth weight as a covariable in the statistical model was significant (*P* < 0.01) for weaning weight and milk DM (kg) intake. Breed of calf was significant (*P* < 0.05) for calf birth weight, hip height, and health cost. Season was significant (*P* < 0.05) for weaning weight total gain, ADG, health cost, average cost per day, average cost per kilogram of gain, and kilograms (DM) of milk consumed.

Results for preweaning and weaning body measurements across feeding treatments for the organic heifer calves are given in Table 1. Birth weight was not different for treatment groups. Weaning weight, hip height, and heart girth were not different (*P* > 0.05) for the WM calves compared with the MR calves. Furthermore, the ADG to weaning of the WM calves was similar (*P* > 0.05) to that of the MR calves. The interpretation of ADG in the current study may be limited because only weaning weight and birth weight were used to determine ADG. These results were inconsistent with those of Godden et al. (2005), who reported that calves fed pasteurized nonsaleable milk had greater weaning weights, weight gain, and ADG than calves fed commercial milk replacer that were fed from 3.8 to 5.6 L of milk or milk replacer per day. Furthermore, Moallem et al. (2010) reported weaning weights were 3.1 kg greater and ADG was 10% greater for calves fed whole milk (8.97 L/d; 1.09 kg of DM/d) versus milk replacer (9.78 L/d; 1.19 kg of DM/d). Shivley et al. (2018) reported that preweaning calves fed (mean = 7 L/d) either pasteurized or unpasteurized milk had greater ADG than calves fed milk replacer. For the current study, the MR (Organi-Calf Instant Milk, Milk Specialties Global) contained higher levels of vitamins A, D_3_, and E than standard milk replacers in the United States; it may thus have provided a more complete nutritional profile to the calf through an improved immune system, and thus similar growth rates to calves fed WM. Milk intake on a DM basis was greater (*P* < 0.05) for MR calves than for WM calves. However, the 2.34-kg difference across the 56 d of the study may not be biologically meaningful and was slightly greater than the standard error of the means. The day-to-day variability in nutrient content (fat and protein) of total solids in the WM may have reduced the differences between WM and MR feeding. Yoho et al. (2017) reported that total solids in whole milk may vary by 7% on farm, concluding that WM is a highly variable source of nutrition for calves and that pasteurized WM may not provide low bacteria counts in milk. Gain-to-feed and feed-to-gain ratios were not different between treatment groups.

**Table 1:** Least squares means and standard errors of means for preweaning body and health measurements, weaning body measurements, and milk feeding behaviors of organic dairy calves by feeding treatment^1^

Measurement	Whole milk (n = 40)		Milk replacer (n = 41)	
	LSM	SE	LSM	SE
Birth weight (kg)	36.86	0.85	38.05	0.85
Weaning weight (kg)	79.4	2.48	77.86	2.47
Weaning hip height (cm)	91.84	1.21	91.09	1.20
Weaning heart girth (cm)	100.90	1.36	101.05	1.36
Total gain (birth to weaning; kg)	40.72	2.48	39.17	2.47
ADG (kg/d)	0.73	0.04	0.70	0.04
Milk intake (kg, DM basis)	48.56^a^	1.89	50.90^b^	1.89
Gain-to-feed ratio	0.84	0.02	0.77	0.02
Feed-to-gain ratio	1.24	0.04	1.32	0.04
Fecal score	0.88^a^	0.08	1.54^b^	0.08
Visit duration (min/visit)	2.4^a^	0.2	3.01^b^	0.2
Drinking speed (mL/min)	1,301.4^a^	1.2	581.0^b^	1.2

a,b Means within a row without common superscripts are different at *P* < 0.05.

1 Reported means and SE are based on feeding treatment averages during the preweaning period.

The milk feeding allowance of 8 L/d in the current study was greater than that in previously reported studies, and therefore may have contributed to the lack of differences observed in the current study. Feeding programs with increased rates of WM or MR have gained in popularity over the past 10 yr, and the increased growth rates were consistent with increased milk feeding rates (Kertz et al., 2017). Perhaps feeding of calves on an automated calf feeder with smaller meals per day contributed to the similar growth rates compared with slug feeding of calves twice per day on US dairy farms. Sharon et al. (2020) concluded that challenges for calves fed a high plane of nutrition may be reduced by increasing the number of feeding times per day, which automated feeders facilitate with less labor requirements. The calves in the current study were allotted 8 L of whole milk or milk replacer per day, which is greater than the amount fed on the average dairy farm in the United States. Most US dairy farms feed, on average, a total of 5.7 L of milk per day, with 2.6 L of milk per feeding (Urie et al., 2018). Regardless, ADG in the current study was similar to that of Holstein calves from a national survey (0.73 kg/d; Urie et al., 2018), and all calves achieved greater than 1.5 times their birth weight at 60 d of age. The lack of differences between treatment groups in the study for growth was unexpected. However, growth rates may have been similar because all calves in the current study were fed a higher plane of nutrition compared with calves on the average US dairy farm. Growth rates of calves may be influenced by health events, fat and protein contents in the WM or MR, lactose content or fatty acid profile of the WM and MR, adequate and correct pasteurization of milk, calf starter protein concentration, and calf starter consumption by calves. Fat and protein contents of the WM in the current study varied from 4.25 to 4.35% and from 3.25 to 3.43%, respectively. Therefore, all of these factors may have played a role in the lack of differences observed for growth in the current study. Given the ADG results observed in the current study, calves were meeting their energy needs.

Results for fecal scores across feeding treatments for calves are also given in Table 1. Calves fed MR had greater (*P* < 0.05) fecal scores than calves fed WM. It is possible that calves fed MR had higher fecal scores because of the greater percent solids in the liquid diet fed to the calves. However, the higher fecal scores could also be associated with greater nutrients consumed from either MR or calf starter for the MR calves. Furthermore, the whole milk likely contained significantly higher levels of immunoglobulins and other immune factors than the milk replacer (Foley and Otterby, 1978). Jorgensen et al. (2017) reported that automated feeders require regular maintenance to ensure proper mixing of milk replacers. Further, they reported that bacterial contamination was common in the milk replacer storage area of the automated feeder. It is possible that the milk replacer was not mixed adequately or that bacterial contamination was present in the milk replacer storage area, which could have exacerbated the incidence of scours in the current study.

Results for drinking behaviors across feeding treatments for organic dairy calves are given in Table 1. The WM calves had shorter (*P* < 0.05) visits to the feeding station than did MR calves. The drinking speed of WM calves was greater (*P* < 0.05) than that of MR calves. Knauer et al. (2017) found that calves fed whole milk drank at an average speed of 877 mL/min, which was lower than that of the WM calves in the current study. However, the current study had a higher milk allowance per day and higher total solids for WM and MR, which may have affected the differences observed between studies for drinking speed. Furthermore, the brand of automated calf feeder was different between studies, which might have affected drinking speed of calves. Taste preference of calves may have also played a role in drinking speed; perhaps calves did not like the taste or texture of the milk replacer dispensed. Competition among calves in pens may have contributed to the difference observed between treatments for drinking speed. Calves were more likely to be denied milk at a feeding station visit; therefore, when milk was available, a calf might be more likely to drink more quickly. Therefore, the visit duration of a calf is likely related to drinking speed.

Least squares means for health cost, cost of WM or MR, and economic analysis of WM and MR for the 2 feeding treatments during the preweaning period are summarized in Table 2. As expected, WM calves had lower (*P* < 0.05) total milk feed costs during the preweaning period compared with MR calves, and the average cost per day was lower (*P* < 0.05) for WM calves than for MR calves. The average cost per kilogram of gain for the WM calves was also significantly lower (*P* < 0.05) than for the MR calves. The profitability results are similar to those reported by Godden et al. (2005), who found total savings of $34 per calf from birth to weaning when feeding milk compared with milk replacer, an economic advantage of $0.69 per calf per day compared with milk replacer. In the current study, calves fed MR had $88 greater feed costs than calves fed WM. Therefore, the cost was $1.59 more per day to feed preweaning calves MR compared with WM. The cost to feed WM calves in the current study was greater than the cost to feed organic calves fed 5 L/d in a group-fed system ($187/calf; Bjorklund et al., 2013), but similar to the cost to feed organic calves fed 6 L/d of milk once ($255/calf) or twice ($266) per day (Kienitz et al., 2017). Regardless, housing system (individual, group-housed, automated feeder) and milk feeding method and quantity may play an important role in calf growth and health and profitability of organic dairy calves (Pempek et al., 2013, 2017).

**Table 2:** Least squares means and standard errors of means for health cost, whole milk or replacer cost, and economic analysis of milk and milk replacer for during the first 8 wk of life^1^

Measurement	Whole milk		Milk replacer	
	LSM	SE	LSM	SE
Health cost ($)	4.65	1.03	5.88	1.01
Milk feed cost ($)	245.57^a^	11.50	333.55^b^	11.43
Average cost/day ($)	4.47^a^	0.19	6.06^b^	0.19
Average cost/gain ($/kg)	6.39^a^	0.28	8.83^b^	0.28

a,b Means within a row without common superscripts are different at *P* < 0.05.

1 Reported means and SE are based on feeding group averages during the preweaning period.

Differences in growth rate and health of calves may not have been observed in the study because of some limitations of the study. Starter grain consumption was not recorded for individual calves because the calves were raised in groups and starter consumption would be difficult to measure in calves fed using an automated calf feeder. However, systems must be evaluated to accurately record individual grain intake for calves on automated calf feeding systems. Automated grain feeding systems are available for use on dairy farms; however, these systems were not installed at the research farm in the current study. Calf starter has a large effect on nutrient consumption and growth of calves. Group size of calves may affect growth rates of calves in an automated feeding system. Larger groups may increase competition of calves for the nipple feeding station because older calves push small calves out of the nipple feeding station (Medrano-Galarza et al., 2018). However, companies that manufacture automated feeding system have recently configured the software for feeders to allow for all nipple feeding stations to feed calves, which was not the case in the current study. Results of the research may not be applicable to all organic dairy farms because calves raised on specific farms may be affected by differences in colostrum management, breed of calf, housing system, disease prevalence on farm, and milk feeding allowance.

Our results showed that organic dairy heifer calves fed an organic milk replacer had similar weight gains and body dimensions to calves fed organic whole milk. Based on the results of this study, organic dairy producers may achieve adequate BW gain when feeding 8 L/d of a 20% protein and 22% fat organic milk replacer to feed calves. However, it was more expensive to feed organic calves MR compared with WM, which may affect the profitability of organic dairy farms. Successful and profitable management of dairy calves is of critical importance to an organic dairy. Therefore, producers must focus on all aspects of calf management to maintain growth rates, minimize health problems, and ensure profitability for the success of the dairy.

## References

[bib1] Bjorklund E.A., Heins B.J., Chester-Jones H. (2013). Whole-milk feeding duration, calf growth, and profitability of group-fed calves in an organic production system. J. Dairy Sci..

[bib2] Foley J.A., Otterby D.E. (1978). Availability, storage, treatment, composition, and feeding value of surplus colostrum: A review. J. Dairy Sci..

[bib3] Godden S.M., Fetrow J.P., Feirtag J.M., Green L.R., Wells S.J. (2005). Economic analysis of feeding pasteurized nonsaleable milk versus conventional milk replacer to dairy calves. J. Am. Vet. Med. Assoc..

[bib4] Hansen L.B. (2000). Consequences of selection for milk yield from a geneticist's viewpoint. J. Dairy Sci..

[bib5] Heinrichs A.J., Jones C.M., Gray S.M., Heinrichs P.A., Cornelisse S.A., Goodling T.C. (2013). Identifying efficient dairy heifer producers using production costs and data envelopment analysis. J. Dairy Sci..

[bib6] Jorgensen M.W., Adams-Progar A., de Passillé A.M., Rushen J., Godden S.M., Chester-Jones H., Endres M.I. (2017). Factors associated with dairy calf health in automated feeding systems in the Upper Midwest United States. J. Dairy Sci..

[bib7] Kertz A.F., Hill T.M., Quigley J.D., Heinrichs A.J., Linn J.G., Drackley J.K. (2017). A 100-Year Review: Calf nutrition and management. J. Dairy Sci..

[bib8] Kienitz M.J., Heins B.J., Chester-Jones H. (2017). Growth, behavior, and economics of group-fed dairy calves fed once or twice daily in an organic production system. J. Dairy Sci..

[bib9] Knauer W.A., Godden S.M., Dietrich A., James R.E. (2017). The association between daily average feeding behaviors and morbidity in automatically fed group-housed preweaned dairy calves. J. Dairy Sci..

[bib10] Littell R.C., Henry P.R., Ammerman C.B. (1998). Statistical analysis of repeated measures data using SAS procedures. J. Anim. Sci..

[bib11] McGuirk S.M. (2018). https://www.vetmed.wisc.edu/fapm/svm-dairy-apps/calf-health-scorer-chs/.

[bib12] Medrano-Galarza C., LeBlanc S.J., DeVries T.J., Jones-Bitton A., Rushen J., de Passillé A.M., Endres M.I., Haley D.B. (2018). Effect of age of introduction to an automated milk feeder on calf learning and performance and labor requirements. J. Dairy Sci..

[bib13] Moallem U., Werner D., Lehrer H., Zachut M., Livshitz L., Yakoby S., Shamay A. (2010). Long-term effects of ad libitum whole milk prior to weaning and prepubertal protein supplementation on skeletal growth rate and first-lactation milk production. J. Dairy Sci..

[bib14] Pempek J.A., Eastridge M.L., Botheras N.A., Croney C.C., Bowen Yoho W.S. (2013). Effects of alternative housing and feeding systems on the behavior and performance of dairy heifer calves. Prof. Anim. Sci..

[bib15] Pempek J.A., Schuenemann G.M., Holder E., Habing G.G. (2017). Dairy calf management—A comparison of practices and producer attitudes among conventional and organic herds. J. Dairy Sci..

[bib16] SAS Institute (2018).

[bib17] Sharon K.P., Hulbert L.E., Davis E.M., Ballou M.A. (2020). The effects of plane of milk replacer nutrition on the health, behavior, and performance of high-risk Holstein bull calves from a commercial calf ranch. Appl. Anim. Sci..

[bib18] Shivley C.B., Lombard J.E., Urie N.J., Kopral C.A., Santin M., Earleywine T.J., Olson J.D., Garry F.B. (2018). Preweaned heifer management on US dairy operations: Part VI. Factors associated with average daily gain in preweaned dairy heifer calves. J. Dairy Sci..

[bib19] Urie N.J., Lombard J.E., Shivley C.B., Kopral C.A., Adams A.E., Earleywine T.J., Olson J.D., Garry F.B. (2018). Preweaned heifer management on US dairy operations: Part I. Descriptive characteristics of preweaned heifer raising practices. J. Dairy Sci..

[bib20] USDA-AMS (Agricultural Marketing Service) (2021). National Organic Program. https://www.ams.usda.gov/rules-regulations/organic.

[bib21] Yoho W.S.B., Hansen C.M., Stephas E.L., Rao M.M.R., Earleywine T.J., Van Roekel L.J., Radmer M.J., Miller B.L. (2017). Variation of nutrient content and bacteria count of pasteurized waste milk fed to dairy calves. J. Dairy Sci..

[bib22] Zwald A., Kohlman T.L., Gunderson S.L., Hoffman P.C., Kriegl T. (2007).

